# What Are Patients Saying About Minimally Invasive Spine Surgeons Online: A Sentiment Analysis of 2,235 Physician Review Website Reviews

**DOI:** 10.7759/cureus.24113

**Published:** 2022-04-13

**Authors:** Justin Tang, Christopher A White, Varun Arvind, Samuel Cho, Jun S Kim, Jeremy Steinberger

**Affiliations:** 1 Orthopedic Surgery, Icahn School of Medicine at Mount Sinai, New York, USA; 2 Neurological Surgery, Mount Sinai Health System, New York, USA

**Keywords:** natural language processing, online reviews, patient satisfaction, sentiment analysis, minimally invasive

## Abstract

Objective

Physician review websites are becoming increasingly popular for patients to find and review healthcare providers. The goal of this study was to utilize quantitative analyses to understand trends in ratings and written comments on physician review websites for Society of Minimally Invasive Spine Surgery (SMISS) members.

Methods

This is a cross-sectional study. The reviews of SMISS surgeons were obtained from healthgrades.com, and sentiment analysis was used to obtain compound scores of each physicians' reviews. SMISS surgeons who were international or had fewer than three written reviews, often consisting of residents and fellows, were excluded. Inferential statistics were utilized, and word frequency analysis reported the phrases used to characterize reviews.

Results

One hundred sixty-nine surgeons met the inclusion criteria. 98.6% were males and the mean age was 51.7 years old. A total of 2,235 written reviews were analyzed. Younger surgeons were significantly more likely to receive higher star ratings (p<0.01). Positive behavioral characteristics, such as “kind” and “bedside manner,” conferred significantly improved odds of receiving positive reviews (p<0.01). Ancillary “staff” conferred a 2x greater odds of receiving a positive review whereas a comment on “wait” times halved a surgeon’s odds (p<0.01). Sentences describing pain drove down the odds of positive reviews whereas those describing pain relief produced greater odds of positive reviews (p<0.01).

Conclusion

Physicians who were younger, personable, provided sufficient pain relief, and who worked in favorable offices received the most positive reviews. This study informs SMISS members on the traits deemed important by patients who ultimately review surgeons online.

## Introduction

Minimally Invasive Spine Surgery (MISS) is quickly becoming a staple within the orthopedic and neurosurgical fields. Current trends in MISS have seen an increase in its use over the last few decades, and minimally invasive surgery is the fastest growing market segment within spine surgery [1-2]. MISS techniques are being used for fusion, decompression, and tumor excision surgeries throughout the spine [1,3]. With further implementation being expected as technology continues to advance, it becomes necessary that spine surgeons understand how both MISS techniques and surgeons are being perceived by the patients who undergo these procedures. A paucity of literature has analyzed patients' conceptions of MISS. However, in the limited available literature, it has been shown that patients generally have positive perceptions surrounding MISS techniques and perhaps a tendency to desire MISS compared to more traditional options (i.e., open surgery) [4-6]. These recent findings make it imperative that we further understand patients' perceptions of the surgeons who perform these procedures and what factors lead to a patient experience that is both satisfying and fulfilling.

Alongside the growth of MISS has been the growth of patient utilization of online physician review websites, a trend especially seen in the orthopedic field [6]. Patients can rate, review, and comment on their overall medical experiences with specific providers through these online platforms. Analyzing the information deposited on these websites has become an important subject within the spine community. Eight individual studies have been published in just the last three years [7-14]. Surface confidence, trustworthiness, and likeability were generally associated with positive reviews, while scheduling difficulties, increasing surgeon age, and long wait times resulted in more negative remarks [8,10,12].

A more narrowed analysis of physician review websites has shown these trends mentioned above for various national spine societies, including the Scoliosis Research Society (SRS), Cervical Spine Research Society (CSRS), and North American Spine Society (NASS) [7-14]. However, to date, an analysis of physician review websites for MISS surgeons, in particular, has not been conducted but would stand to be a valuable addition to the current literature on patients' perceptions surrounding MISS. This subset analysis of spine surgeons would provide important insights for the MISS community and influence the patient-centered care surgeons strive to provide. Thus, the goal of this present study was to utilize quantitative analysis to understand trends in both ratings and written comments left on physician review websites for the Society of Minimally Invasive Spine Surgery (SMISS) members. We hypothesize that younger SMISS surgeons who display trustworthiness and confidence will receive the highest remarks like the spine mentioned above. At the same time, poor ancillary environments and long wait times will drive down overall surgeon ratings.

## Materials and methods

Data Acquisition

A list of SMISS surgeons was obtained from the SMISS society. Initially, this list had 325 members in the society; however, this number was lower than that used in the study as those without profiles (usually residents or international providers) were removed. Surgeons were excluded if they did not have profiles or had fewer than three written reviews. The written and star-rating reviews of SMISS surgeons were obtained online from healthgrades.com, which provides publicly available data. All reviews up through September 2021 were included. Healthgrades.com was chosen because it was one of the first websites to appear for any given provider upon searching for providers online.

Additionally, many websites have firewalls preventing large data scraping, but healthgrades.com was one of the few that permitted web scraping of large amounts of data from the website without restriction. For the rest of the study, star-rating reviews refer to the online ratings out of five stars given to surgeons. The star-rating reviews utilized in this study provide an overall average star rating for each surgeon.

Sentiment analysis

The analysis in this project was made possible by using the "Valence Aware Dictionary and sEntiment Reasoner" (VADER) sentiment analysis package. This is a standard python toolkit that can provide sentiment analysis for written prose. [15] It takes in sentences and outputs a sentiment score on a continuous scale from -1 to +1. This score thus represents how positive or negative a sentence is depending on the given sentiment. VADER was utilized to obtain the scores of all reviews for SMISS surgeons.

VADER score calculation

VADER was developed and trained on a dictionary of words rated independently by 10 human trainers. These individuals were tested for inter-rater reliability and provided a score for every word of the dictionary. Their original scores ranged from -4 to +4 to indicate how positive or negative a given the word in the dictionary is [15]. This established program can then be augmented by inputting target sentences and paragraphs. The program takes in all of the sentences of a given review and scans for given words found in the VADER dictionary. It then calculates and normalizes the scores to between -1 to +1. The program can also modulate the score given changes based on punctuation, capitalization, and adverbs used to modulate words. For example, if the word "very" were used prior to a positive word (such as "helpful"), it would cause the score to be closer to +1 than if "helpful" was used alone. The contrary is also true where if the word "not" is utilized before "helpful," the contribution to the overall score would cause it to go closer to -1. As a result, a score closer to -1 would mean the overall review and paragraphs have negative sentiment, and scores closer to +1 represents more positive sentiment. In social media, punctuation, and capitalization are often used for emphasis. As a result, VADER can also account for and factor in excessive punctuation and capitalization. For example, if a sentence reads "He is great!!!!," the exclamation marks would augment the score to be even closer to +1 than if "He is great" was written by itself. 

Model validation

Linear regression analysis was implemented to compare the average sentiment analysis score to each physician's average reported five-point star score. This was performed to show the relationship between our calculated sentiment analysis scores and the online reported out of 5 stars scores. 

Data analysis

Student t-tests were used to assess gender-related to average sentiment scores of written reviews, and linear regression was used for age. Additionally, we performed a word frequency analysis to see what words were being utilized most often in the best and worst reviews. Due to the score distributions, as most reviews were largely positive, a positive review for the word frequency analysis was defined as an overall score >0.75, and a negative review was defined as a review with a negative sentiment analysis score. Further, bigrams (two-word strings) were also analyzed for their frequencies in these reviews to provide greater context for the words being seen in the frequency analysis. Bigrams refer to these two-word sequences such as "no pain". Finally, we performed a multiple logistic regression on keywords and word pairs to analyze their association with a sentiment score > 0.50.

## Results

Surgeon demographics

One hundred sixty-nine SMISS surgeons' profiles were analyzed following the inclusion and exclusion criteria, including 2,235 reviews. All of the demographic characteristics of the surgeons were also extracted from healthgrades.com (Table 1). The physician's age and gender identity were pulled directly from what was reported online. Some physicians did not have their gender or age listed; these grades were not included in respective analyses.

**Table 1:** Physician demographics

Demographics	Counts
Gender	
Male	144 (98.6%)
Female	2 (1.4%)
Age	Mean age = 51.7
<40	17 (13.2%)
40-49	45 (34.9%)
50-59	42 (32.6%)
>60	25 (19.4%)

Model validation: Linear regression

**Figure 1:**
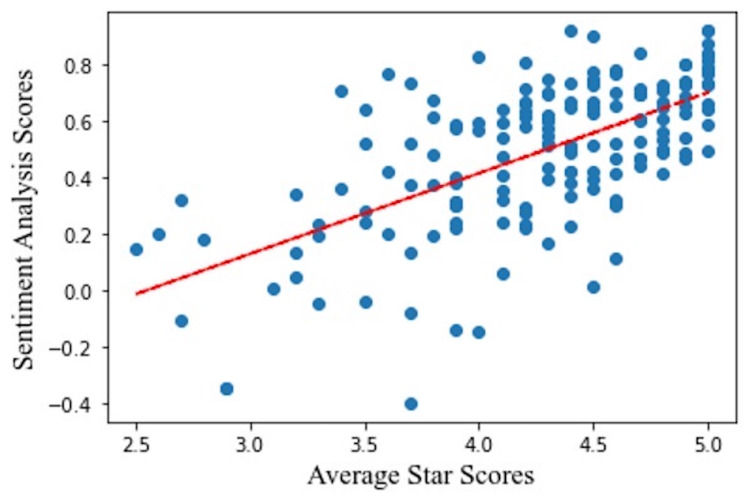
Linear regression model comparing average reported online star scores to calculated sentiment analysis scores

The linear regression analysis of average sentiment analysis scores to average star scores showed a positive correlation between the scores (r2 = 0.63, p<0.01), indicating that the calculated sentiment analysis scores correlate significantly with the online reported values (Figure 1).

Model validation and demographic analysis: Student T-test and Linear regression

**Figure 2:**
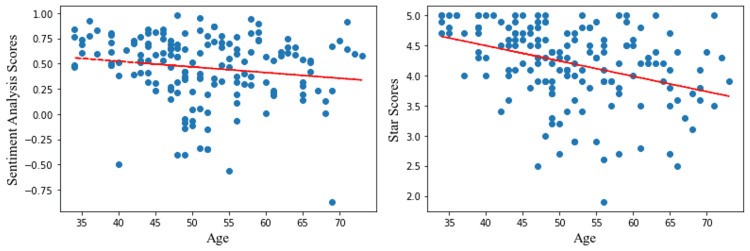
Linear Regression Analyses for Sentiment Analysis and Star Scores Compared to Physician Age

A Student t-test was performed to check for a significant difference between the means of the sentiment analysis scores given to male and female surgeons. The test indicated no significant correlation between gender and greater or lower sentiment analysis scores or star scores. The linear regression indicates that younger surgeons had significantly lower star scores (r^2^= -0.37, p<0.01) and lower sentiment scores (r^2^= -0.16,p=0.04). These results are summarized in Table 2 and Figure 2.

**Table 2:** Student T-test comparing Star and Written Reviews to Gender

	Male Average	Female Average	P Val
Written Reviews	+0.43	+0.42	0.50
Star Reviews	4.25	4.19	0.80

Word frequency analysis

Frequencies of most used words recognized by NLTK are also reported. The most frequently used and meaningful words used to describe top-rated surgeons are words correlating with pain, care, kindness, and friendliness. The worst reviews often use pain, rude, and numb words (Table 3). The reported words are only those that were clinically or behaviorally relevant. All other words, such as descriptors, were removed to focus on characteristics that would help determine what factors affect patient reviews. For example, words such as "amazing" and "bad" were removed. Although they describe the patient's experience, they do not aid in analyzing what factors contribute to the reviews themselves.

**Table 3:** Clinically-Relevant Single Word Frequency Analysis of Best and Worst Reviews

Best Reviews		Worst Reviews	
Word	Frequency	Word	Frequency
Pain	593	Pain	423
Care	325	Problem	67
Caring	216	Care	30
Kind	130	Rude	26
Friendly	112	Numbness	15

In order to provide greater context to the words found in the word frequency analysis, a bigram analysis was also performed to look for the most commonly used word pairs. This was to determine whether the context of the words was affecting the score, pushing words with negative or positive connotations in the other direction. For example, pain in the positive reviews did not intuitively make sense at first. In the bigram analysis of the most positive review, the most frequently-used two-word sequence that was clinically/behaviorally relevant was "pain-free," thus confirming our hypothesis that the context had changed. "No pain" was the second most used and relevant bigram. The other three referred to more behavioral aspects (Table 4). These findings confirm the suspicions that the words preceding and following pain were affecting their connotation and thus appropriately being scored as positive attributes for the providers.

**Table 4:** Clinically-Relevant Bigram Frequency Analysis of Best and Worst Reviews

Best Reviews		Worst Reviews	
Bigram	Frequency	Bigram	Frequency
Pain-free	98	No pain	54
No pain	42	Back pain	42
Kind caring	40	Pain-free	32
Cares Patients	36	Severe pain	30
Feel Comfortable	33	Lower back	22

For the most negatively reviewed surgeons, their descriptors centered around behavioral aspects and levels of pain and numbness. Pain seems to be a larger contributing factor as "pain" was utilized 423 times, whereas the first instance of a behavioral attribute, "rude," was only used 26 times (Table 3). A bigram analysis was also performed to confirm that the pain contributed to negative sentiment in this context. Bigram analysis of negative reviews also supports this claim as all of the top 5 clinically relevant and highest frequency bigrams were about pain, descriptors of pain, or regions of pain (Table 4). This indicates that pain is a clear driver of negative reviews.

Multiple logistic regression

Finally, a multiple logistic regression was performed on clinically relevant keywords. This regression indicates how specific high frequency or clinically relevant words or phrases influence the odds of receiving a sentiment score >0.50 (Table 5). A review with a score >0.50 indicates that the review is largely positive for a particular surgeon.

**Table 5:** Multiple logistic regression analysis on clinically relevant keywords

	2.5% CI	97.5% CI	OR	P val
Approachable	0.13	10.89	1.19	0.88
Wait	0.30	0.97	0.54	0.04
Pain	0.27	0.44	0.35	<0.01
No pain	0.20	0.95	0.44	0.04
Severe pain	0.10	0.85	0.29	0.02
Pain free	0.75	2.69	1.42	0.29
Relief	1.17	6.09	2.67	0.02
Staff	1.64	2.86	2.16	<0.01
Confident	0.69	3.17	1.48	0.32
Listens	0.94	5.50	2.28	0.07
Bedside manner	1.54	10.30	3.98	<0.01
Knowledgeable	0.82	2.70	1.49	0.19
Kind	1.48	5.32	2.80	<0.01
Home	0.55	2.33	1.13	0.74
Same day	0.24	1.96	0.68	0.47
Cost	0.01	2.02	0.17	0.16
Incision	0.11	1.48	0.41	0.18
Discharge	0.03	7.84	0.49	0.61
Swelling	0.15	13.17	1.42	0.76
Muscle	0.18	15.37	1.68	0.64
Recovery time	0.18	14.45	1.63	0.66
Recommend	2.02	3.42	2.63	<0.01
Long surgery	0.03	7.84	0.49	0.61
Pain medication	0.05	1.33	0.26	0.10

A "kind" provider had a significant 2.80 odds ratio of receiving a positive review, and the "bedside manner" of the surgeon conferred a 3.98 odds of review. However, pain, descriptors, and wait time negatively affected the odds of receiving a positive review. "Pain," "No pain," and "Severe pain" all decreased the odds to 0.35, 0.44, and 0.29, respectively, and a "wait" decreased the odds to 0.54. Ancillary factors such as "staff" also affected the reviews, as the inclusion of "staff" had a 2.16 odd of a positive review.

## Discussion

The field of Minimally Invasive Spine Surgery (MISS) is seeing drastic growth in annual utilization and surgical technology [1,2,16,17]. However, a paucity of literature has analyzed the patient perception of MISS and MISS surgeons and what factors in a patient encounter matter most. This latter point becomes increasingly important when one considers that patients often select their healthcare providers based on recommendations and reputation and are increasingly using the internet. This study expands upon the current literature by analyzing patient-reported comments on SMISS surgeons online. Using sentiment analysis via a machine learning algorithm, we analyzed the star-rating and written reviews of 169 SMISS members. We identified multiple clinical, personal, and ancillary characteristics used to describe the most positively and negatively rated surgeons. As this analysis can be conducted in real-time, it proves to be a valuable tool capable of quantifying dynamic patient comments across various physician review websites.

Multiple previous studies have quantitatively analyzed physician review websites for spine surgery societies (i.e., NASS, CSRS, SRS) [7-14]. In each of these studies, surgeon age was negatively correlated with overall ratings [7,9,14]. In the present study, we too report that younger surgeons reported significantly higher scores than older SMISS surgeons (p<0.01). We also note that compared to the previous physician review website analyses of spine surgeons (i.e., NASS and SRS), the SMISS surgeons' ages were, on average, two years younger, possibly related to the novelty of MISS [7,9,10,13]. Further, previous spine literature has reported no significant differences in the ratings received by spine surgeons based on surgeon gender [11,13,14].

Similarly, we report that qualitative and quantitative reviews do not differ between male and female SMISS members; however, this study is underpowered due to low female contingency. The aforementioned spine society members were also primarily male [11,13,14]. Across major orthopedic and neurosurgical societies, it is estimated that only 5% of the American Academy of Orthopaedic Surgeons (n=26,914) and 6% of the American Association of Neurological Surgeons (n=4,178) members are female physicians [18,19]. In order to foster inclusion and diversity throughout neurosurgical and orthopedic practices, we strongly believe that an important consideration for all spine societies is the narrowing of the present gender gap.

In this study, being "kind," "caring," and "thoughtful" correlated with strong patient sentiment and appreciation of minimally invasive surgeons. Those described as "kind" had a 2.8x odds of receiving a positive comment. Comments with the term "listens" embedded also trended towards significance with a 2.3x odds of a positive review. Interestingly, a "confident" surgeon did not significantly increase the surgeon's odds of receiving a positive review. From this analysis, it is clear that the personality traits exhibited by the surgeons play a vital role in the experiences of their patients. While of utmost importance, being a technically proficient surgeon is not sufficient in and of itself; it is necessary to practice proper bedside manners. Further, SMISS members with largely positive reviews were 2.6x more likely to be "recommend[ed]" by their patients on these online profiles (p<0.01). Thus, physicians who exemplify more of these qualities are more likely to bolster their appearances online, which encourages more patients to be confident in selecting them as a provider.

This analysis shows that, while being "kind," "caring," and "thoughtful" correlated with strong patient sentiment and appreciation of a minimally invasive surgeon, the most impactful factor in patient sentiment was pain and pain resolution. The pain was the most critical hit word in both positive (593 times) and negative reviews (423 times). The former is focused on pain resolution as elucidated by the positive review bigram frequency analysis, and the latter usually is in the context of lasting or residual pain. All of the top five bigrams in the negative reviews are focused on pain and its regions. Patients often come into clinics with preconceived notions about pain resolution. Surgeons should spend time managing pain expectations and setting proper and realistic expectations. "Numbness" was also a common complaint within negative SMISS reviews. Numbness often takes a longer time than radiculopathy to resolve, and in some cases of severe nerve injury, numbness will persist after surgery. Like pain expectation management, proper counseling about numbness resolution is also a facet of patient care that SMISS surgeons should devote time and attention to.

Further, reviews of clinical reports indicate similar clinical outcomes for MISS and open surgery patients who underwent lumbar fusion and decompression surgeries [20-22]. Despite this, patients have been shown to perceive decreased postoperative pain levels as a significant advantage of MISS. In a study by Narain et al., 84% of patients seeing a spine surgeon thought MISS would be less painful than open spine surgery [5]. This may result from literature suggesting that MISS results in less pain immediately following the surgery than open spine surgery, as is emphasized by reductions in narcotic use and length of stay [23-25]. In our analysis of patient comments specifically for minimally invasive spine surgeons, our single-term, bigram, and multiple logistic regression results also indicate "pain" as the primary driver of patient comments. For example, patients who mentioned "pain-free" or "relief" in their comments resulted in a 1.4x (p<0.01) and 2.7x (p=0.02) higher chances of a surgeon receiving a positive sentiment score. Further, any mention of "pain" meant that surgeons had a significantly reduced chance of receiving a positive review (0.4x; p<0.01).

Moreover, there is a major emphasis in the current literature on the advantages and disadvantages of MISS and more conventional open spine surgery. In general, MISS is lauded for providing patients with shorter hospitalization times, smaller incisions (i.e., minimal scarring), reductions in postoperative opioid usage, and less peripheral tissue damage than open spine surgery for the treatment of the same conditions [23-26]. Recent literature also suggests that MISS can incur similar or reduced healthcare costs for patients due to the associated decreases in lengths of stay compared to open spine surgery [26,27]. At the same time, there are also significant drawbacks to MISS, including increased radiation exposure, which is technically more challenging for the surgeon comparatively [21,28]. Patients' perceptions of minimally invasive techniques further highlight perceived reductions in recovery time, complication rates, and cost as key advantages for MISS. At the same time, decreases in intraoperative visibility are seen as a potential disadvantage in MISS [4,5]. However, there was a notable discrepancy in comparing the aforementioned patient-perceived pros and cons of surgery with our sentiment analysis. We report no significant influence of phrases such as "incision", "discharge", "same day", "cost", "recovery time", or "long surgery" on a SMISS member's overall sentiment score. This finding indicates that while MISS has intrinsic advantages and disadvantages, these characteristics are not being stressed at the patient level for online reviews. However, this could also be since the majority of MISS surgeons also offer traditional surgical approaches. Nevertheless, we still believe that it is a worthwhile endeavor for SMISS surgeons to stress the qualities that make minimally invasive techniques unique in the preoperative and postoperative patient evaluations. These reviews are the information that patients can publicly obtain online. From this analysis, however, it appears as though ancillary, clinical, and surgeon personal characteristics drive sentiment scores rather than select MISS-specific attributes.

Following the present study's findings, we believe that MISS surgeons would benefit from spending more time addressing preoperative concerns and the pain expectations of their patients. Despite the current literature on the perceptions of MISS as a field, it is not what the patients are writing about in their reviews of providers. Instead, the emphasis is directed at the care received at the facility and the outcomes of their procedures. As a result, it would be prudent for MISS surgeons to dedicate more time to counseling patients on their postoperative expectations. Patients may perceive surgery as a "magic bullet" that guarantees a certain level of pain and symptom resolution that is not necessarily certain with surgery. By quelling fears and addressing any misconceptions before operating, surgeons may be able to improve their patients' perceptions if not all of their expectations have been met. 

There are several limitations to this study. Using only SMISS members in the present analysis, we could not assess the online comments for all MISS surgeons. Additionally, with only two female surgeons included in this analysis, we could not definitively determine if a member's gender had any influence on their sentiment scores. Moreover, using publicly available data meant we could not determine a patient's motivation for their comments or the time at which they commented on a SMISS member following their healthcare visit or operation. This aforementioned point leads to inherent sampling bias. Nevertheless, given the current trends in MISS case volume, reported cost reductions, and surgeons becoming more comfortable using MISS techniques, it is imperative that we fully understand patient sentiments with MISS surgeons as this form of spine surgery will continue to increase in popularity. This present analysis provides a platform by which online patient comments can be continually examined and reviewed.

## Conclusions

This study is the first machine learning algorithm analysis of online reviews of minimally invasive spine surgeons. We identified demographic, personal, and clinical indications of positive and negative reviews for SMISS surgeons. While it is vital for minimally invasive spine surgeons to be both personable and technically skilled, we identified pain relief and pain management as the primary drivers of sentiment scores. Given that patients utilize the internet to both find and recommend healthcare providers, the findings in this study should encourage physicians to review how they present pain expectations at each patient encounter and prioritize top-quality bedside manner.
